# C—H Bond Activation Mechanism by a High‐Valent Dinuclear Copper Complex: Unraveling the Effect of a Base by a Theoretical Study

**DOI:** 10.1002/jcc.70070

**Published:** 2025-02-20

**Authors:** Oceane Mangel, Helene Jamet

**Affiliations:** ^1^ CNRS Department of Molecular Chemistry (DCM, UMR 5250) Université Grenoble Alpes Grenoble France

**Keywords:** copper complexes, DFT and DLPNO‐CCSD(T) calculations, HAA mechanism, intrinsic bond orbitals

## Abstract

Recently, an electrochemically monooxidized dinuclear copper(II) complex [Cu_2_(L)(*μ*‐OH)_2_]^2+^ with the dipyridylethane naphthyridine ligand (L) has been shown to activate the recalcitrant aliphatic C_sp_^3^—H bond of toluene (bond dissociation free energy, BDFE = 87.0 kcal mol^−1^) at room temperature. The mechanistic pathway turns from stoichiometric to catalytic upon addition of a base (2,6‐lutidine), suggesting a modification of the reactive species. Herein, we report theoretical calculations to characterize the reactive species and obtain a detailed understanding of the reactivity. Since different electronic structures are possible for these high valent systems, we perform DFT calculations coupled to CCSD(T) ones using the DLPNO‐CCSD(T) scheme. Our results show that the presence of a base will impact the nature of the reactive species but also the type of mechanism involved in the C—H activation.

## Graphical abstract

A theoretical study to understand at a molecular level the activation of strong C—H bonds by high‐valent dinuclear copper complexes is reported using investigation of the mechanistic pathways with the intrinsic bond orbital (IBO) analysis and evaluation of accurate electronic energies with DFT and DLPNO‐CCSD(T) calculations.

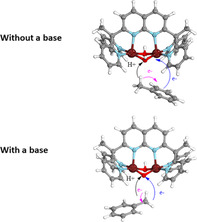

## 1 Introduction

The oxidation/oxygenation of alkanes with high bond dissociation free energy (BDFE) by energy‐efficient, environmentally benign, and cost‐effective catalytic devices is a formidable challenge for our near‐future society [1]. Those reactions are particularly important in the context of global warming, where finding ways to reduce the atmospheric concentration of strong greenhouse gases such as methane is essential [2]. This makes catalysts using environmentally friendly metals such as copper of great interest [3], especially as several copper enzymes such as the particulate Methane Mono‐Oxygenase (pMMO) are able to activate strong C‐H bonds easily, including the one of methane. For this enzyme, a theoretical study has postulated that a mixed‐valent Cu^II^‐Cu^III^/O_2_ cluster in the active site could be the oxidizing intermediate in the catalytic cycle [4]. However, despite numerous studies and subsequent x‐Ray structures [5], the debate over the identity and the nuclearity of the active center stays open. Consequently, challenges remain to explore such mixed‐valent species and their capabilities as efficient intermediates for strong C—H bond activation, as pointed out by Kieber‐Emmons [6].

**FIGURE 1:**
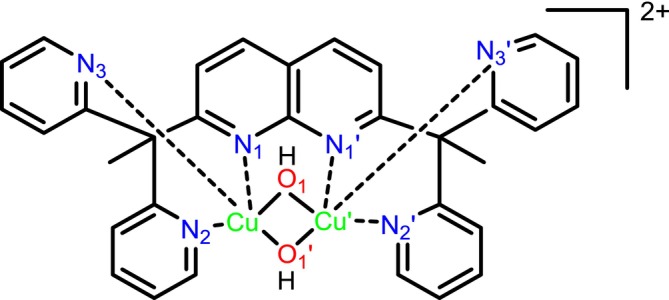
The cationic part *1* ^*2*+^ [Cu_2_(L)(*μ*‐OH)_2_]^2+^ with the dipyridylethane naphthyridine (L) ligand.

In this context, Belle et al. synthesized the dinuclear copper(II) complex *1* [Cu_2_(L)(*μ*‐OH)_2_](ClO_4_)_2_.6H_2_0 with the dipyridylethane naphthyridine (L) ligand for which the cationic part *1* ^*2*+^ is represented in Figure 1 [7]. They showed that the electrochemical mono‐electronic oxidation of the cationic part of *1* in CH_3_CN leads to the formation of a mixed‐valence Cu^II^‐Cu^III^ species, the cationic part *1* ^*3*+^ [Cu_2_(L)(*μ*‐OH)_2_]^3+^. They also demonstrated by electrochemistry in a new publication [8] the capacity of this species to oxidize toluene in acetonitrile solution at room temperature with a pathway that turns from stoichiometric to catalytic upon the addition of a relatively weak base (lutidine, p*K*_a_ = 14.16). A possible *μ*‐oxido *μ*‐hydroxido Cu^II^‐Cu^III^ species is proposed as a transient active species, associated with the deprotonation of one hydroxido bridge of *1* ^*3*+^. In this work, we used computational approaches to understand the reaction mechanism and to characterize the reactive species in the absence or in the presence of a base. Two reactive species were considered: the *1* ^*3*+^ [Cu_2_(L)(*μ*‐OH)_2_]^3+^ species and in the presence of a base, its basic form *1‐H* ^*2*+^ [Cu_2_(L)(*μ*‐O)(*μ*‐OH)]^2+^. DFT calculations were done to determine the geometry, the electronic structure, and the spin ground state of these species. However, the open‐shell electronic configurations of these oxido and hydroxido dicopper species could result in a strong electron correlation, making it challenging for DFT calculations. To describe the difficulties associated with the computation of the electronic structure of different Cu_2_O_2_ isomers, Cramer et al. coined the term “torture track” [9]. Over the last decades, several computational studies have been done on these systems [10, 11, 12, 13, 14]. In particular, the effects of the percentage of the Hartree‐Fock exchange in the DFT functionals have been evaluated [15]. Therefore, to determine the spin ground state of our reactive species, different functionals have been tested. Moreover, CCSD(T) calculations using the DLPNO‐CCSD(T) scheme [16] were performed in addition to DFT ones. This method is a highly accurate and computationally efficient method for treating electron correlation in large systems, and recently some works [17, 18] have shown its suitability to describe systems involving transition metals with moderate multireference character. Therefore, this method was also used to determine the spin ground state of our two reactive species, and the results were compared to those obtained by DFT calculations. Then this methodology was used to compute electronic energies along the reaction paths to understand the reactivity of these systems with toluene and predict potential system intercrossings.

C—H bond activation involves the transfer of both a proton (H^+^) and an electron (e^−^). Understanding the mechanistic details of these transfers is essential for the design of new systems. When the electron and the proton are transferred in one single elementary step, the process is referred to as a concerted proton and electron transfer (CPET) [19]. This mechanism can be described in two limiting cases, with the electron and the proton transferred together to a single site in a process called H‐atom transfer (HAT), or the electron and proton transferred to two different sites in a concerted proton coupled electron transfer process (cPCET) [20, 21, 22, 23, 24]. Thus, in this work, to obtain a detailed understanding of the reactivity, in complement to the determination of the reaction paths and the evaluation of the energetic barriers, an analysis of the electronic structure along the reaction path was done using the intrinsic bond orbital (IBO) localization scheme of Knizia and co‐workers [25]. It has been demonstrated that changes of IBOs along the reaction coordinate allow the monitoring of electrons through the pathway, which makes it efficient in differentiating between the cPCET or HAT processes associated with a C—H bond‐cleavage reaction [26].

## 2 Computational Methods

Full geometry optimizations and frequency calculations were carried out for all systems using the Gaussian 16 package [27] and the hybrid meta‐GGA functional TPSSh [28] in combination with the def2‐TZVP basis set on copper atoms and the def2‐SVP one on the remaining atoms. The accuracy of this basis set family was shown to be robust in a lot of works, and in particular, in the original work of Weigend and Ahlrichs [29]. The TPSSh functional was chosen for its ability to determine the transition state of the reaction. Among the three functionals tested (PBE0 [30], TPSSh, B3LYP [31]), TPSSh and B3LYP allowed the localization of a transition state, but only the TPSSh functional gave an intrinsic reaction coordinate (IRC) connecting the transition state to the reactant. The IEFPCM [32] solvent model was used for all calculations with the acetonitrile solvent. An “ultrafine” grid was used for numerical integration in DFT and a “fine” grid for the solvent. This protocol validated on the molecular structure in the solid state of the cationic part *1* ^*2*+^ (Figure 1) was also used to compute the reaction free energies associated with the deprotonation of *1* ^*3*+^ and *1* ^*2*+^ by lutidine. Different spin states were considered, including a broken symmetry formalism [33] computed from the triplet spin state using the guess = mix keyword when necessary. In order to obtain accurate electronic energy calculations, single‐point energy calculations were also performed using the def2‐TZVP basis set for all atoms and three hybrid functionals (TPSSh, PBE0, B3LYP). Comparisons were then made using DLPNO‐CCSD(T) calculations [16] on small models of the complexes with the Orca 5.0.4 software [34]. Since a small modification of geometry has a high impact on the energy of these species, the comparison must be done using the same geometry for all systems. Therefore, the coordinates of all atoms of these small models were the same as those of their associated complexes, except for the position of the new hydrogen atoms, which were optimized. The DLPNO‐CCSD(T) calculations were performed with the default NormalPNO settings starting from a TPSSh reference determinant using the larger def2‐TZVPP basis set for Cu, the def2‐TZVP for O, C, N, the def2‐SVP for H, and the corresponding auxiliary def2‐TZVPP/C basis set for Cu, def2‐TZVP/C for O, C, and N, and def2‐SVP/C for H. The SMD [35] solvation model for the solvent with acetonitrile was used. To choose these thresholds and basis sets, a calibration, given in Table S1, was conducted on the relative energy between the doublet and the quartet spin states of 1^3+^.

For analyzing reaction path, DFT intrinsic reaction coordinate (IRC) [36] calculations were carried using the HPC [37] algorithm and frequency calculations on the optimized TS with the “RCFC” option for 100 steps in both directions. For orbital localization using the IBO scheme, single‐point calculations were reperformed along the IRCs with Orca 5.0.4 and the TPSSh functional. The D3(BJ) correction [38] was added and SCF convergence tolerance was set to “TightSCF.” Calculations were speed‐up by using the RIJCOSX approximation using the def2/J universal Coulomb‐fitting basis sets [39]. IBOs were generated using IboView program [40] with “iboexp = 2”. Orbitals and spin densities were visualized with the software VMD [41] with an isovalue equal to 0.04 a.u.

## 3 Results and Discussion

### 3.1 Characterization of the Reactive Species

**FIGURE 2:**
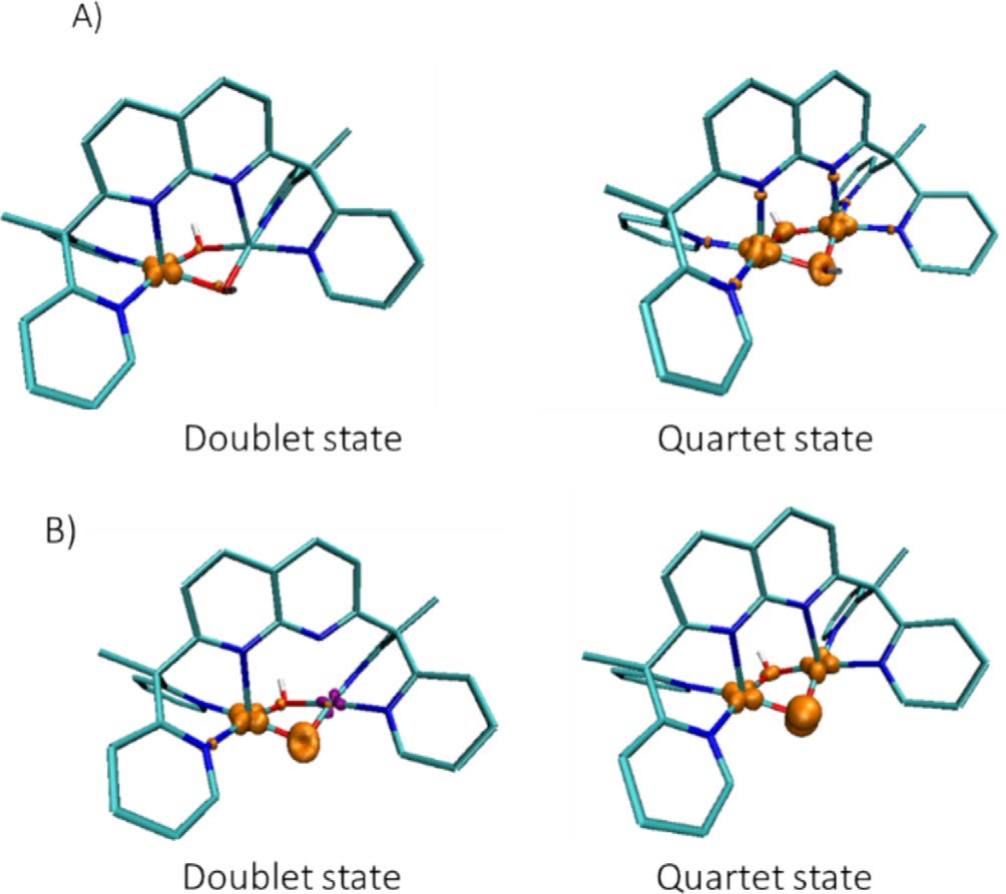
Spin density plot (isosurface equals to 0.04 a.u) for (A) the doublet and the quartet spin state of *1* ^*3*+^ [Cu_2_(L)(*μ*‐OH)_2_]^3+^. (B) The doublet and the quartet spin state of *1‐H* ^*2*+^ [Cu_2_(L)(*μ*‐O)(*μ*‐OH)]^2+^.

DFT calculations were performed to optimize the geometry of the reactive species using the functional TPSSh, the def2‐TZVP basis set on copper atoms and the def2‐SVP one on the remaining atoms. To validate this methodology, we used the crystallographic structure of the cationic part *1* ^*2*+^ (Figure 1), which keeps its geometry upon dissolution in acetonitrile with two Cu atoms at the oxidation state +II in a square pyramidal geometry [7]. Geometry optimizations of *1* ^*2*+^ were performed in the triplet state and using the broken symmetry formalism. For this broken‐symmetry singlet state, we obtained an *S* ^2^ value equals to 1.0045, which is exactly the average between the value of the singlet and the triplet state. Bond distances with copper atoms are given in Table 1. The same geometry is obtained for the two spin states, with a very weak difference of energy equal to 0.5 kcal mol^−1^ in favor of the broken symmetry state. Thus, even if this functional and basis sets are not sufficient to differentiate the two spin states, the perfect agreement between their geometries and experimental data shows the success of this methodology to optimize the geometry of these systems. The *1* ^*3*+^ species, formed after the mono‐electronic oxidation state of *1* ^*2*+^ has an odd number of electrons. Therefore, a doublet and a quartet spin state were used to optimize the geometry of *1* ^*3*+^. The doublet spin state is found as the spin ground state with a difference of energy equal to 5.4 kcal mol^−1^. Figure 2A displays Mulliken spin density plots for the two spin states. For the doublet state, the spin density is localized on one copper atom only, in agreement with the formation of a mixed‐valence species Cu^II^‐Cu^III^, suggesting that the oxidation of the dinuclear copper (II) complex *1* ^*2*+^ occurs on one Cu atom. On the contrary, for the quartet state, the spin density is delocalized on copper, oxygen and nitrogen atoms. Mulliken spin densities are given in Table S2. For the doublet state, these values confirm the oxidation of one copper atom, with values of spin densities equal to 0.60 for Cu and 0.01 for Cu′. For the quartet state, values are equal to 0.70 for Cu, Cu′, 0.34 for O1, O1′, 0.13 for N2, N3, N2′, N3′, and 0.15 for N1, N1′, suggesting a symmetrical structure for *1* ^*3*+^ in the quartet state. The main distances of the optimized geometries given in Table 1 confirm this result, with a symmetrical structure on the two copper atoms for the quartet spin state and an asymmetrical structure for the doublet spin state. Bond distances around the oxidized copper atom, distances with Cu′ in red in Table 1, decrease for the doublet spin state, to the exception of the distance between Cu′ and N1′, which increases slightly. An analysis of the orbitals of the broken symmetry state of *1* ^*2*+^ suggests that the removed electron is located onto an antibonding orbital (Figure S1). The important difference of energy between the doublet and the quartet spin states, added to the fact that EPR experiments at 100 K indicates the formation of a localized mixed‐valent Cu^II^‐Cu^III^ species for *1* ^*3*+^ [7], allow us to be confident on the determination of the spin ground state that is the doublet spin state, for this system.

**TABLE 1:** Bond distances with copper atoms for the different complexes studied, notations of atoms are given in Figure 1.

Selected bond distances (Å)	1^2+^ BS/triplet (RX)	1^3+^ Doublet/quartet	1‐H^2+^ Doublet/quartet	TS‐1^3+^ doublet	TS‐1‐H^2+^ doublet/quartet
Cu…Cu′	2.78/2.78 (2.79)	2.83/2.81	2.72/2.71	2.86	2.71/2.72
Cu‐O1	1.97/1.97 (1.96)	2.00/1.94	2.03/1.98	1.94	1.98/1.97
Cu‐O1′	1.97/1.97 (1.95)	2.00/1.94	1.89/1.90	2.07	1.96/1.95
Cu‐N1	2.27/2.27 (2.29)	2.21/2.11	2.25/2.20	2.22	2.26/2.26
Cu‐N2	2.00/2.00 (1.98)	1.97/1.99	2.00/2.01	1.99	2.01/2.01
Cu‐N3	2.00/2.00 (2.00)	1.97/1.99	2.00/2.00	2.00	2.02/2.01
Cu′‐O1	1.97/1.97 (1.96)	1.86/1.94	1.89/1.98	1.94	1.95/1.97
Cu′‐O1′	1.97/1.97 (1.95)	1.86/1.94	1.80/1.90	2.05	1.88/1.95
Cu′‐N1′	2.27/2.27 (2.29)	2.29/2.11	2.32/2.20	2.23	2.32/2.25
Cu′‐N2′	2.00/2.00 (1.98)	1.89/1.99	1.95/2.01	1.99	1.99/2.01
Cu′‐N3′	2.00/2.00 (2.00)	1.89/1.99	1.92/2.00	1.98	1.97/2.01

*Note:* Bond distances in red are associated to the Cu^III^ for the doublet spin state of *1* ^*3*+^ and *1‐H* ^2+^.

**FIGURE 3:**
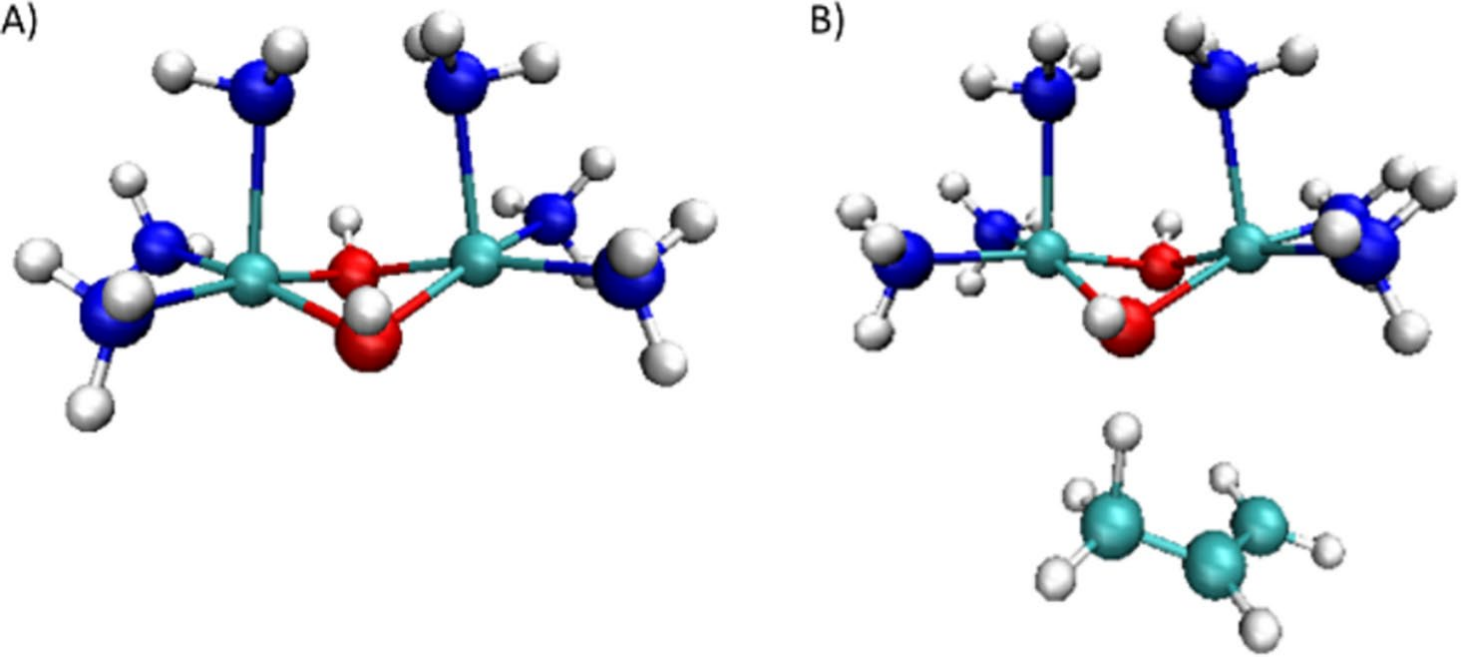
Small models used for DLPNO‐CCSD(T) calculations. Energies calculations were done on the optimized geometries of the doublet or the quartet spin state. (A) Small model for *1* ^*3*+^. (B) Small model for *TS‐1* ^*3*+^.

The same study was done for the reactive species formed in the presence of a base. As for *1* ^*3*+^, the geometries of the quartet or the doublet spin state of the complex *1‐H* ^*2*+^ [Cu_2_(L)(*μ*‐O)(*μ*‐OH)]^2+^ were optimized, and the optimized geometry of the quartet or the doublet spin state of *1* ^*3*+^ in which one hydroxido bridge has been deprotonated was used as starting structures. The analysis of the spin density and the geometry (Figure 2B, Table 1) gives an asymmetrical structure associated with a mixed‐valent Cu^II^‐Cu^III^ species for the doublet spin state and a symmetrical structure for the quartet spin state, as it was the case for the doublet of the *1* ^*3*+^ species. However, compared with the electronic structure of *1* ^*3*+^, the spin density of the quartet spin state shows a high localization on the *μ*‐oxido group, O1′, as pictured in Figure 2B, suggesting an electronic structure close to a radical oxygen atom and two Cu^II^, noted Cu^II^‐O^**˙**^‐Cu^II^. Mulliken spin densities given in Table S2 confirm these results, with a value equal to 1.08 on O1′ for the quartet spin state, and a very weakly negative value on Cu′ equal to −0.10 for the doublet spin state. The difference in energy between the doublet and the quartet spin state of *1‐H* ^*2*+^ is small, with a value equal to 1.2 kcal mol^−1^ in favor of the doublet spin state. Single‐point energy calculations on these two optimized geometries were done with two other well‐known hybrid functionals, the B3LYP and PBE0 functionals (Table S3). The percentage of Hartree–Fock exchange of these functionals is 10% for TPSSh, 20% for B3LYP, and 25% for PBE0. Energies of the two spin states remain close, but the trend is reversed in favor of the quartet spin state, in agreement with the increase of the Hartree–Fock exchange known to stabilize high‐spin states [42]. As no experimental data for this transient species are available, it is difficult to conclude on the nature of the spin ground state. Therefore, DLPNO‐CCSD(T) calculations were performed in addition to DFT calculations. As a reference, single‐point energy calculations were first performed on the *1* ^*3*+^ species, using the small model of the complex given in Figure 3A, in the symmetrical and asymmetrical optimized geometries associated with the doublet and quartet spin states. We computed a high difference in energy between the two spin states equal to 19.2 kcal mol^−1^ in favor of the doublet state. Values of the diagnostic T1 are equal to 0.0172 for the doublet state and 0.0197 for the quartet state. These small values confirm the single‐reference character in the wavefunction of our species and the suitability of this method to describe these species. Compared to DFT calculations with the functional TPSSh, the relative energy between the doublet state and the quartet state is four times larger (19.2 kcal mol^−1^ against 5.4 kcal mol^−1^), confirming the formation of the mixed‐valent Cu^II^‐Cu^III^ species in agreement with experimental data. Using the same approach for *1‐H* ^*2*+^, a very small difference in energy of 0.15 kcal mol^−1^ in favor of the quartet spin state was obtained. Values of T1 stay small with a value of 0.0184 for the doublet spin state and 0.0233 for the quartet spin state. This result suggests the two spin states are very close in energy and confirms the trend computed using different functionals in Table S3. Thus, in the following, both structures were used to study the reactivity of *1‐H* ^*2*+^ with toluene.

Finally, we computed the reaction free energies associated to the deprotonation of *1* ^*3*+^ by the lutidine to form *1‐H* ^*2*+^. Calculations were done using the functional TPSSh in combination with the def2‐TZVP basis set on copper atoms and the def2‐SVP one on the remaining atoms in agreement with the protocol validated for the geometry optimization of the systems. In considering a doublet spin state for *1* ^*3*+^ and a doublet state or a quartet state for *1‐H* ^*2*+^, we obtain respectively a value equals to −4.7 kcal mol^−1^ and to −5.8 kcal mol^−1^. This result is consistent with the formation of the complex of *1‐H* ^*2*+^ in presence of lutidine in agreement with the hypothesis previously by Belle et al. [8] Another possible hypothesis would be to consider first the deprotonation of *1* ^*2*+^ in presence of lutidine to form the complex [Cu_2_(L)(*μ*‐O)(*μ*‐OH)]^+^, named *1‐H* ^+^, which would then be oxidized to *1‐H* ^*2*+^. To discriminate this hypothesis, we optimize the geometry of *1‐H* ^+^ in the triplet and broken symmetry spin states following the same protocol as described previously. Value of the deprotonation free energies in the triplet spin state is equal to 27.3 kcal mol^−1^, showing that *1‐H* ^+^ does not exist under the experimental conditions described by Belle et al. [8].

### 3.2 Characterization of the Mechanisms in the Presence or in the Absence of a Base

**FIGURE 4:**
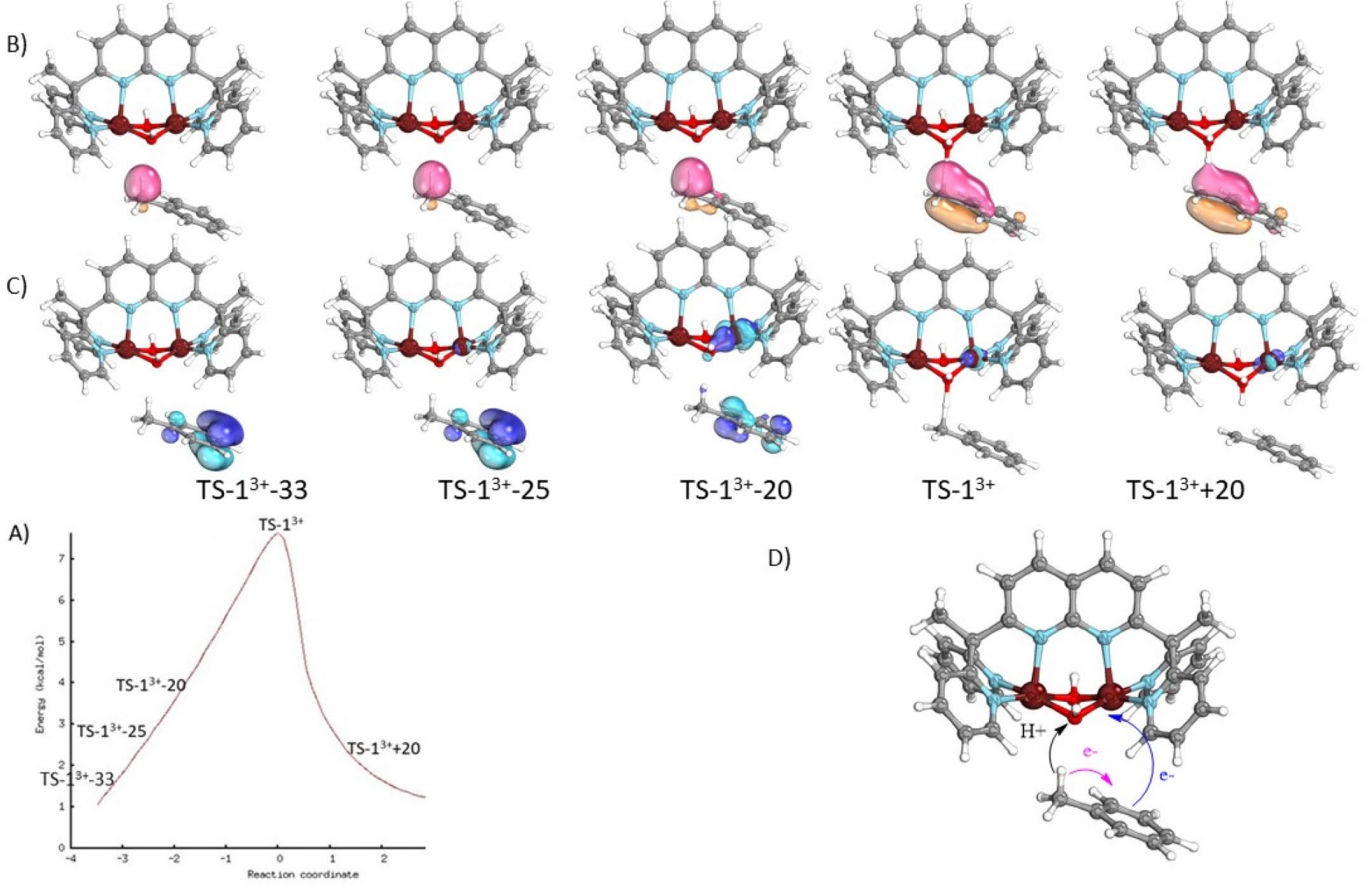
(A) IRC Plot for the doublet spin state of *TS‐1* ^*3*+^ (computed at with the TPSSh functional, the def2‐TZVP basis set on copper atom and def2‐SVP on the remaining atoms). Transformations of the Intrinsic bond orbitals (IBOs) along the IRC: (B) in pink, the α‐IBO of the C—H bond of toluene, (C) in blue, the β‐IBO of the π‐cloud of toluene transformed into a d orbital of the Cu^III^ of 1^3+^.

To study the C—H bond activation of toluene by *1* ^3+^, a mechanism was first explored between a doublet spin state, associated with the Cu^II^‐Cu^III^ electronic structure of *1* ^3+^ and the closed‐shell structure of toluene. A transition state (TS) and the intrinsic reaction coordinate (IRC) connecting to the reactants and products were determined (Figure 4A). The main metrical parameters around copper atoms for the TS, noted *TS‐1* ^*3*+^, are given in Table 1 and compared to those of the doublet spin state of *1* ^*3*+^. Bond distances around the oxidized copper atom of *1* ^*3*+^, corresponding to distances with Cu′ in red in Table 1, increase de novo for *TS‐1* ^*3*+^. The asymmetric structure Cu^II^‐Cu^III^ of *1* ^*3*+^ is lost, suggesting an electron transfer on the Cu^III^. IBOs were computed along the IRC. In particular, changes of IBOs for α‐ and β‐electrons of C‐H bond cleavage from toluene to *1* ^*3*+^ along the IRC have been carefully analyzed. Evolution of the α‐IBO of the C—H bond is shown in Figure 4B, in pink. It remains localized on the toluene substrate after the proton transfer. Similar evolution is obtained for the corresponding β‐IBO (Figure S2). Therefore, the electron transfer associated with completing the hydrogen transfer must take place from another IBO. Figure 4C gives this orbital in blue. It is a β‐IBO of the π‐cloud of toluene which evolves into a d orbital of the copper atom before *TS‐1* ^*3*+^. This orbital reflects the transfer of a π electron from the toluene towards the Cu^III^ of *1* ^*3*+^. Then, since the transfer of the proton and the transfer of the electron involve different donor/acceptor centers, as it is illustrated in Figure 4D, this process is best described as a cPCET pathway. Moreover, we see that the electron transfer occurs between the frames *TS‐1* ^*3*+^ *‐25* and *TS‐1* ^*3*+^ *‐20* of Figure 4A before the transition state *TS‐1* ^*3*+^ associated with the proton transfer.

**FIGURE 5:**
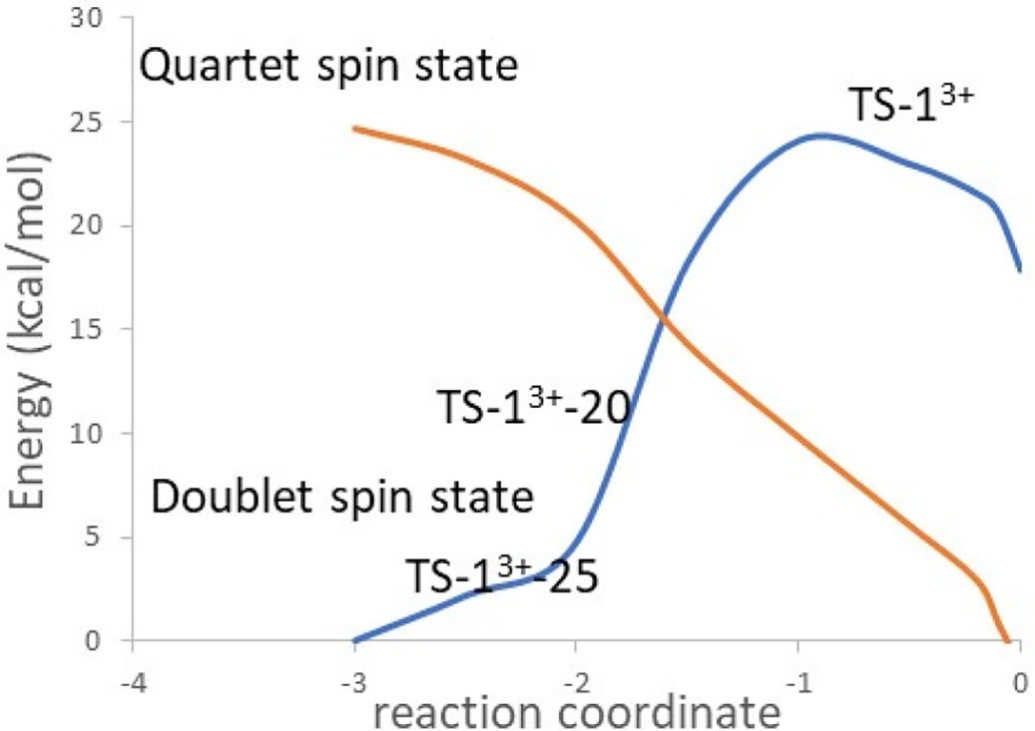
Single point energy DLPNO‐CCSD(T) calculations on the frames of the IRC given in Figure 4A both in the doublet (in blue) and in the quartet (in orange) spin states.

However, since the reactive species evolves into two Cu^II^ for *1* ^*3*+^ and one radical for the toluene along the IRC, we cannot exclude the possibility of a system intercrossing between the doublet and the quartet spin states. Indeed, multiple spin‐state scenarios have already been published to describe the reactivity of transition metal complexes [43, 44, 45], making it important to consider different spin states during the exploration of the mechanism. Single point energy calculations on the geometry of *TS‐1* ^*3*+^ were performed both in the quartet and the doublet spin states using the same approach described before with DLPNO‐CCSD(T) calculations. The small model used to describe this structure is given in Figure 3B. A high difference of energy between the two spin states is obtained, equal to 18.1 kcal mol^−1^ in favor of the quartet spin state, confirming a change of the spin state between this structure and the reactants for this reaction. DLPNO‐CCSD(T) calculations were thus performed on all the frames of the IRC (Figure 5) and the system intercrossing was determined to occur just after the electron transfer (frame *TS‐1* ^*3*+^ *‐20* on the Figure 4A), indicating that the transfer of the electron triggers a rearrangement of the system from the doublet to the quartet spin state. This change gives a lower energy pathway with a computed free energy of activation with DLPNO‐CCSD(T) calculations estimated at 14 kcal mol^−1^ compared with one of 24 kcal mol^−1^ when a system intercrossing is not considered (Figure 5).

**FIGURE 6:**
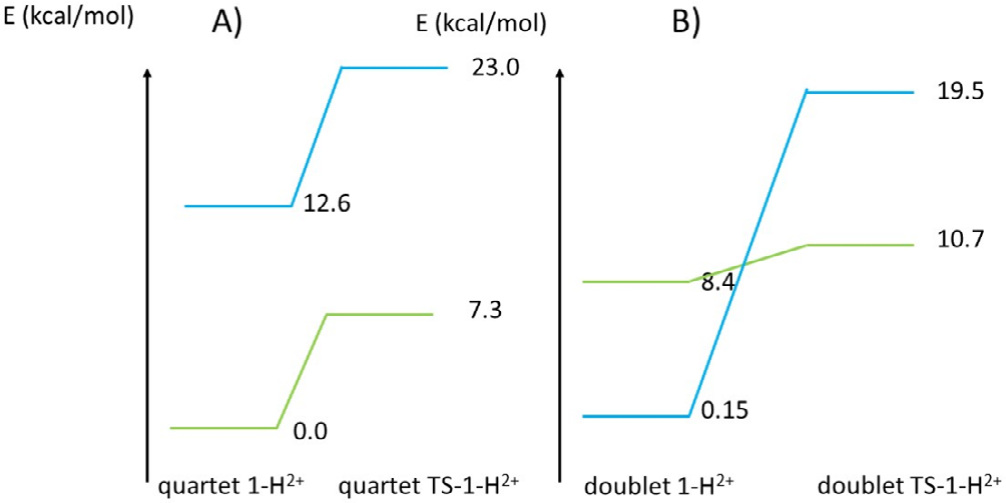
DLPNO‐CCSD(T) calculations: In green energy computed for a quartet spin state, in blue energy computed for a doublet spin state (A) on the optimized geometry of the quartet *TS‐1‐H* ^*2*+^ and *1‐H* ^*2*+^ small models (B) on the optimized geometries of the doublet *TS‐1‐H* ^*2*+^ and *1‐H* ^*2*+^small models.

**FIGURE 7:**
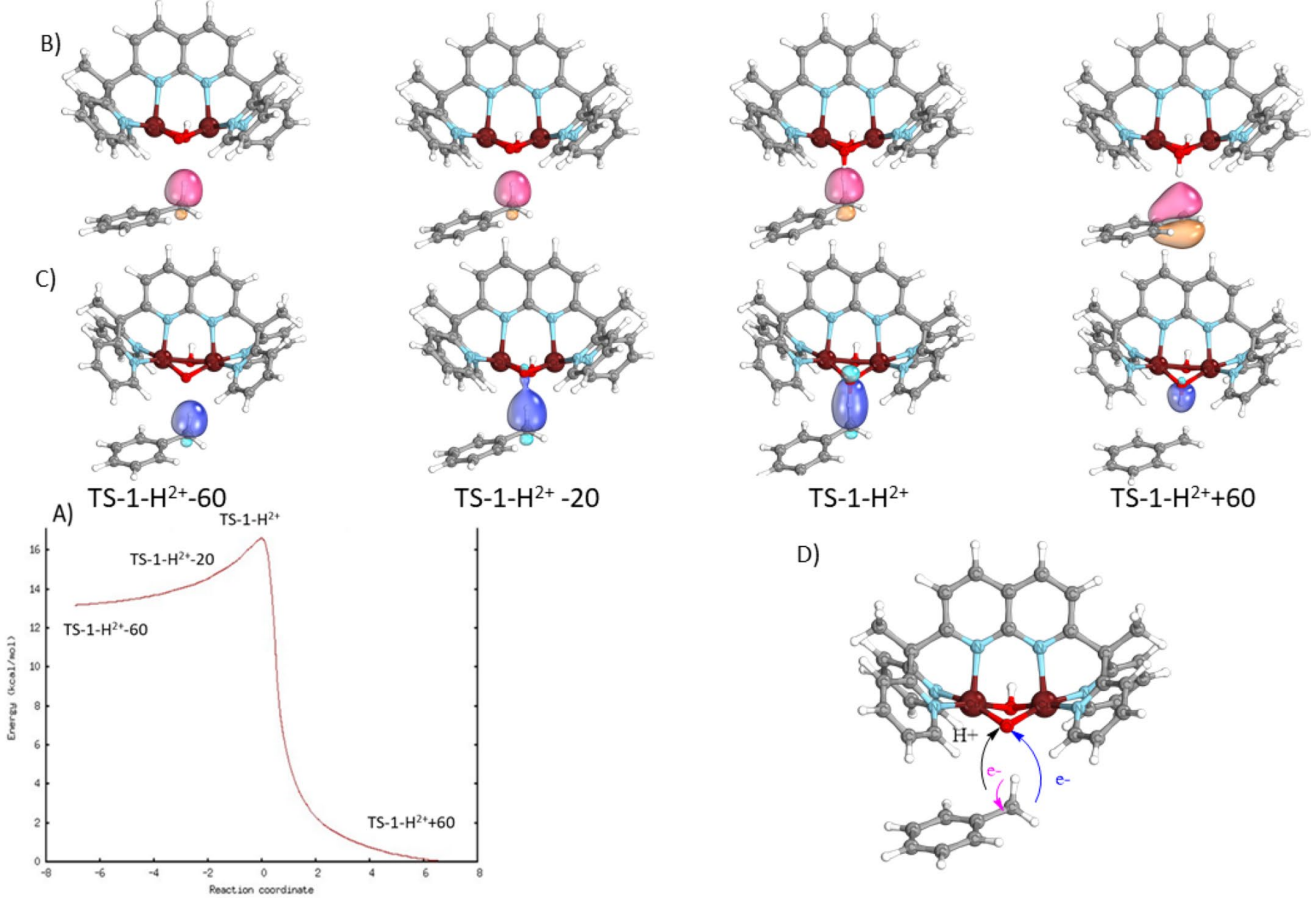
(A) IRC Plot for the quartet spin state of *TS‐1‐H* ^*2*+^ (computed at with the TPSSh functional, the def2‐TZVP basis set on copper atom and def2‐SVP on the remaining atoms). Transformations of the Intrinsic bond orbitals (IBOs) along the IRC: (B) in pink, the α‐IBO of the C—H bond of toluene, (C) in blue, the β‐IBO of the C—H bond of toluene transformed into the new O—H bond of *1‐H* ^*2*+^.

To study the C—H bond activation of toluene by *1‐H* ^*2*+^, the reactive species in the presence of a base, since the difference of electronic energy between the doublet and the quartet spin states of *1‐H* ^*2*+^ is very weak, a transition state (*TS‐1‐H* ^*2*+^ in Table 1) with its intrinsic reaction coordinate (IRC) was determined for both spin states. For the doublet spin state, as for *TS‐1* ^*3*+^, bond distances with the oxidized copper atom of *1‐H* ^*2*+^, Cu′ in red in Table 1 for *1‐H* ^*2*+^, increase de novo for *TS‐1‐H* ^*2*+^, suggesting an electron transfer on the Cu^III^. A free energy of activation equal to 8.6 kcal mol^−1^ is computed using DFT calculations. For the quartet spin state, associated with the Cu^II^‐O^˙^‐Cu^II^ electronic structure for *1‐H* ^*2*+^, the optimized geometry of *TS‐1‐H* ^*2*+^ shows a symmetrical structure with close bond distances with the two copper atoms (Cu and Cu′ in Table 1) and other atoms. The free energy of activation computed using DFT calculations is equal to 4 kcal mol^−1^. DLNPO‐CCSD(T) calculations were also performed to find a possible system intercrossing between the doublet and the quartet spin states for these two paths. The relative energies of the different systems computed with the two spin states using small models of the Figure 3B are plotted in Figure 6. In Figure 6A, calculations were performed on the optimized geometry of the quartet *TS‐1‐H* ^*2*+^ and *1‐H* ^*2*+^ and in Figure 6B on the optimized geometry of the doublet *TS‐1‐H* ^*2*+^ and *1‐H* ^*2*+^. This comparison shows that for this system, the lowest energy pathway involves the quartet *TS‐1‐H* ^*2*+^ associated with the Cu^II^‐O^˙^‐Cu^II^ electronic structure of the quartet *1‐H* ^*2*+^ without the possibility of a system intercrossing (structures in green on the Figure 6A). Therefore, as presented previously, the IRC associated with this pathway and the change of the intrinsic bond orbitals (IBOs) linked to the C—H bond cleavage along this IRC are given in Figure 7. The α‐IBO (Figure 7B, in pink) remains as a radical in the toluene product while the β‐IBO (Figure 7C, in blue) is associated with the transformation of the C—H bond of toluene to the new O—H bond of *1‐H* ^*2*+^. This transformation indicates that the proton and the electron are transferred on the *μ*‐oxido group of *1‐H* ^*2*+^, in agreement with a HAT mechanism (Figure 7D). A low value of the free energy of activation of this reaction is obtained, equal to 7.3 kcal mol^−1^ using DLNPO‐CCSD(T) calculations (Figure 6A), two times lower than that obtained for *1* ^*3*+^ with toluene, estimated previously at 14 kcal mol^−1^. Based on experimental cyclic voltammograms simulations, a rate constant equal to *k*_f_ = 1.1 M^−1^ s^−1^ is obtained for the chemical oxidation of toluene by complex *1* ^*3*+^. Then, when 2,6‐lutidine is added, the reaction becomes catalytic with a significantly higher rate constant with *k*_f_ equal to 7(±1) × 10^5^ M^−1^ s^−1^ [8]. Our results match well with these data, confirming the mechanisms. The presence of a base therefore not only impacts the nature of the reactive species but also the type of mechanism involved in the toluene activation.

## 4 Conclusions

As the interest for systems able to activate strong C—H bonds increases, it is important to find theoretical methods allowing us to characterize the transient active species involved in these reactions properly. In this study, we considered the activation of the aliphatic C—H bond in toluene by the two reactive species formed with the dipyridylethane naphthyridine ligand (L): the species *1* ^*3*+^ [Cu_2_(L)(*μ*‐OH)_2_]^3+^ and the species *1‐H* ^*2*+^ [Cu_2_(L)(*μ*‐O)(*μ*‐OH)]^2+^ obtained after deprotonation of one hydroxido bridge of *1* ^*3*+^ in the presence of 2,6‐lutidine. Since the energies of different spin states are very close for these systems, a reliable protocol using a high level of calculations on small models was considered to characterize them and describe correctly their reaction pathways. Different electronic structures were found for the ground state of the two systems, with a localized mixed‐valent Cu^II^‐Cu^III^ species for *1* ^*3*+^ and a delocalized structure associated with an oxyl Cu^II^‐O^˙^‐Cu^II^ species for *1‐H* ^*2*+^. This change of the electronic structure impacts directly the type of mechanism involved in the reaction. While a cPCET pathway with a high free energy of activation is obtained for *1* ^*3*+^, a HAT mechanism is found for *1‐H* ^*2*+^ with a small free energy of activation. These results, in agreement with experimental data, clarify the relationships between the electronic structures of the copper‐oxygen adducts and their reactivity properties towards alkanes, which is an essential step in order to develop efficient systems.

## Conflicts of Interest

The authors declare no conflicts of interest.

## Supporting information


Data S1.

## Data Availability

Coordinates of the different complexes studied are provided in Supporting Information. Files associated with the IRC paths and some examples of ORCA, CCSD(T), and IBO calculations are available at https://doi.org/10.5281/zenodo.14229333.
