# Supplementary material for: C—H Bond Activation Mechanism by a High‐Valent Dinuclear Copper Complex: Unraveling the Effect of a Base by a Theoretical Study
Source: J Comput Chem. 2025 Feb 20;46(6):e70070. doi: 10.1002/jcc.70070 (PMC11841023; doi:10.1002/jcc.70070)
Supplement: Supplementary file 1 — Data S1. [file JCC-46-0-s001.docx]

Supporting Information

C-H bond activation mechanism by a high-valent dinuclear copper complex: unravelling the effect of a base by a theoretical study

Océane Mangel ^a^, Hélène Jamet ^a*^

[a] Université Grenoble Alpes, CNRS

Department of Molecular Chemistry (DCM, UMR 5250), 38058 Grenoble Cedex 9 (France)
E-mail: [helene.jamet@univ-grenoble-alpes.fr](mailto:helene.jamet@univ-grenoble-alpes.fr)

Table of Content

[1) Calibration of the basis sets and the thresholds associated to the DLPNO-CCSD(T) calculations 3](#_Toc189216287)

[2) Mulliken spin densities 3](#_Toc189216288)

[3) The beta highest occupied orbital of 1^2+^: 4](#_Toc189216289)

[4) Benchmarks of functionals for the relative energy between the doublet and the quartet state of **1-H^2+^** 4](#_Toc189216290)

[5) Evolution of the β-IBO of the C-H bond of toluene along the IRC 4](#_Toc189216291)

[6) Optimized geometries (xyz coordinates) of the different complexes studied 5](#_Toc189216292)

# Calibration of the basis sets and the thresholds associated to the DLPNO-CCSD(T) calculations

This calibration was done in computing the energy of the doublet and the quartet spin states of **1^3+^** using the small model described in Figure 3A of the publication. EPR experiments at 100K have shown the formation of a localized mixed-valent Cu^II^-Cu^III^ species for **1^3+^** (*Inorg. Chem.* **2016**, *55*, 8263) indicating a doublet ground spin state for this reactive species. Three basis sets are tested. The basis set used for the geometry optimization of **1^3+^**, and two larger basis sets. Results are given in the Table S1 and show that the combination of the def2-TZVPP basis set for Cu, the def2-TZVP for O, C, N, the def2-SVP for H with the corresponding auxiliary basis set def2-TZVPP/C basis set on Cu, def2-TZVP/C on O, C, N, and def2-SVP/C on H and the criteria NormalPNO is correct.

|  | The doublet state geometry of **1^3+^** | The quartet state geometry of **1^3+^** |
| --- | --- | --- |
| Cu def2-TZVP, def2-TZVP/C;  H, O, C, N def2-SVP, def2-SVP/C, NormalPNO | 0.0 | 14.3 |
| Cu def2-TZVPP, def2-TZVPP/C;  O, C, N def2-TZVP, def2-TZVP/C,  H ,def2-SVP, def2-SVP/C, NormalPNO | 0.0 | 19.2 |
| Cu def2-TZVPP, def2-TZVPP/C;  O, C, N def2-TZVP, def2-TZVP/C,  H ,def2-SVP, def2-SVP/C, TightPNO | 0.0 | 20.1 |
| Cu def2-QZVPP, def2-QZVPP/C;  O, C, N def2-TZVP, def2-TZVP/C,  H ,def2-SVP, def2-SVP/C, NormallPNO | 0.0 | 19.3 |

Table S1: Basis sets and thresholds tested for DLPNO-CCSD(T) energy calculations (kcal mol^-1^) of the doublet and the quartet spin states of **1^3+^** using the small model described in Figure 3A of the publication.

# Mulliken spin densities

| **Mulliken spin densities** | **1^2+^**  **BS/Triplet** | **1^3+^**  **Doublet/Quartet** | **1-H^2+^**  **Doublet/Quartet** |
| --- | --- | --- | --- |
| **Cu**  **Cu’**  **O1**  **N1**  **N2**  **N3**  **O1’**  **N1’**  **N2’**  **N3'** | **0.59/0.59**  **-0.59/0.59**  **0.00/0.22**  **0.00/0.00**  **0.10/0.10**  **0.10/0.10**  **0.00/0.22**  **0.00/0.00**  **-0.10/0.10**  **-0.10/0.10** | **0.60/0.70**  **0.01/0.70**  **0.08/0.34**  **0.00/0.15**  **0.11/0.13**  **0.11/0.13**  **0.08/0.34**  **0.00/0.15**  **0.00/0.13**  **0.00/0.13** | **0.60/0.60**  **-0.10/0.60**  **0.05/0.26**  **0.01/0.00**  **0.10/0.11**  **0.08/0.08**  **0.32/1.01**  **0.01/0.00**  **-0.02/0.11**  **-0.02/0.08** |

Table S2: Mulliken spin densities of 1^2+^, 1^3+,^ 1-H^2+^, notations of atoms are given in figure 1.

# The beta highest occupied orbital of 1^2+^:


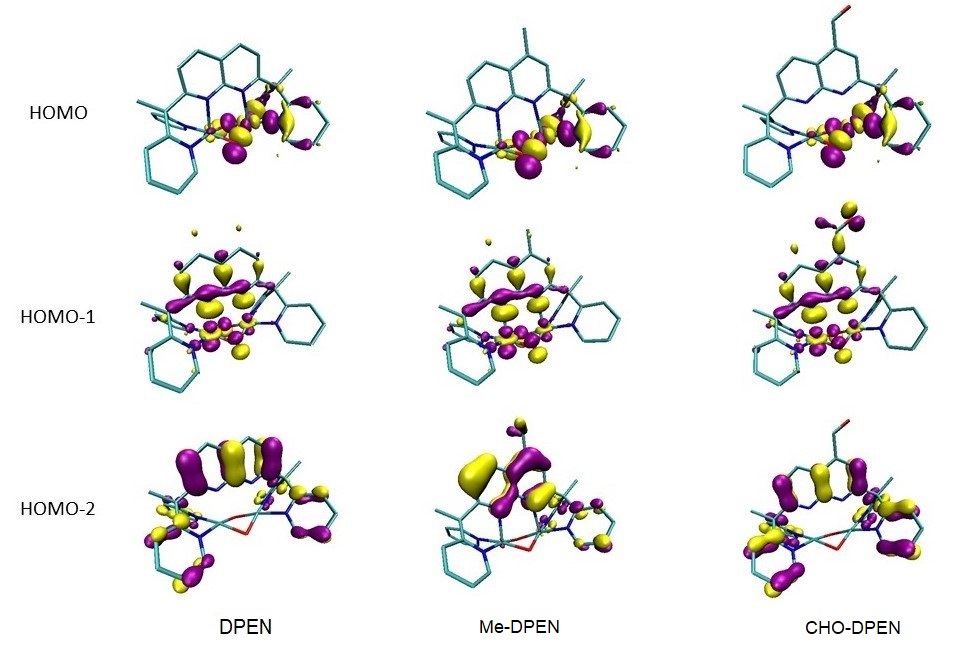


Figure S1: Schematic representation (isosurface is equal to +/- 0.04 a.u) of the beta highest occupied orbital for the broken symmetry **1^2+^,** hydrogen atoms are omitted of the representation.

# Benchmarks of functionals for the relative energy between the doublet and the quartet state of **1-H^2+^**

|  | **TPSSH** | **B3LYP** | **PBE0** |
| --- | --- | --- | --- |
| The doublet spin state geometry of **1-H^2+^** | -1.6 | 2.1 | 4.5 |
| The quartet spin state geometry of **1-H^2+^** | 0.0 | 0.0 | 0.0 |

Table S3: Single point energies computed with three hybrid functionals and the def2-TZVP basis set for all atoms taking the quartet spin state as a reference (kcal mol^-1^). Geometries are optimized using the functional TPSSH, the def2-TZVP basis set on copper atom and the def2-SVP on the remaining atoms.

# Evolution of the β-IBO of the C-H bond of toluene along the IRC

**
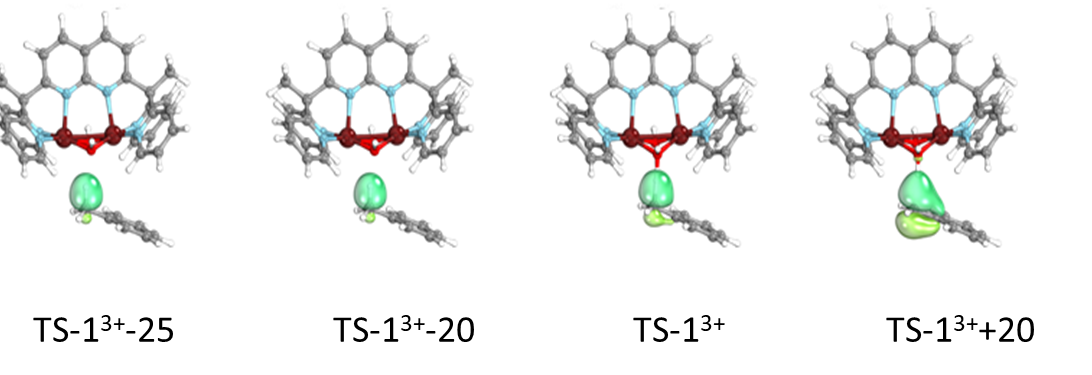
**

Figure S2: Evolution of the β-IBO of the C-H bond of toluene along the IRC associated to the **TS-1^3+^** between **1^3+^** and toluene.

# Optimized geometries (xyz coordinates) of the different complexes studied

| BS 1^2+^ | TS 1^2+^ |
| --- | --- |
| 1 29 0 -1.388439 0.003475 -1.088680  2 29 0 1.388101 0.001921 -1.088805  3 8 0 -0.000914 -1.273133 -1.647166  4 8 0 0.000529 1.281182 -1.640970  5 7 0 -2.804947 -1.391576 -0.885599  6 7 0 -1.174073 -0.001175 1.172421  7 7 0 -2.803382 1.399196 -0.880015  8 7 0 2.803028 -1.394756 -0.886008  9 7 0 2.804653 1.396023 -0.880089  10 7 0 1.173923 -0.002595 1.172318  11 6 0 -4.704767 2.257639 0.257473  12 6 0 -2.406561 -0.004836 3.237232  13 6 0 -2.338428 -0.001830 1.816013  14 6 0 3.761613 -3.463673 -1.615790  15 6 0 -1.243889 -0.007227 3.970763  16 6 0 0.000015 -0.006588 3.299699  17 6 0 -3.726440 1.266748 0.092547  18 6 0 2.406586 -0.008029 3.237021  19 6 0 1.243976 -0.008955 3.970653  20 6 0 -0.000045 -0.003399 1.872511  21 6 0 -4.878848 -0.000599 1.933926  22 6 0 2.813584 2.457697 -1.706452  23 6 0 3.665862 -0.003789 0.985077  24 6 0 -2.813980 -2.449908 -1.716242  25 6 0 -2.811185 2.460768 -1.706521  26 6 0 2.338333 -0.004743 1.815808  27 6 0 -4.725515 -3.355405 -0.608150  28 6 0 -4.706947 -2.252641 0.248915  29 6 0 -3.765935 -3.459498 -1.615035  30 6 0 -3.727732 -1.262033 0.087631  31 6 0 4.878760 -0.006506 1.933504  32 6 0 3.727639 1.262414 0.092383  33 6 0 3.726051 -1.266366 0.087149  34 6 0 -4.722098 3.363748 -0.595304  35 6 0 4.707091 2.252185 0.257372  36 6 0 -3.666028 0.000588 0.985397  37 6 0 -3.762184 3.470874 -1.601558  38 6 0 4.721412 -3.360775 -0.608992  39 6 0 4.704170 -2.258093 0.248205  40 6 0 2.810786 -2.452996 -1.716780  41 6 0 3.765727 3.466719 -1.601429  42 6 0 4.725597 3.358386 -0.595263  43 1 0 -0.001295 -2.057626 -1.074294  44 1 0 0.000963 2.062835 -1.064224  45 1 0 -5.455202 2.180073 1.039795  46 1 0 -3.361350 -0.005327 3.754183  47 1 0 3.741818 -4.305196 -2.308905  48 1 0 -1.270017 -0.009600 5.062171  49 1 0 3.361419 -0.009847 3.753888  50 1 0 1.270197 -0.011495 5.062058  51 1 0 -4.875704 0.884680 2.581535  52 1 0 -4.876551 -0.888266 2.578237  53 1 0 -5.813669 0.000895 1.358609  54 1 0 2.021386 2.481381 -2.457068  55 1 0 -2.022026 -2.470439 -2.467210  56 1 0 -2.019009 2.483461 -2.457191  57 1 0 -5.490211 -4.124507 -0.484266  58 1 0 -5.457143 -2.177417 1.031700  59 1 0 -3.747162 -4.301127 -2.308050  60 1 0 4.875529 -0.894313 2.577623  61 1 0 4.876650 0.878634 2.581300  62 1 0 5.813533 -0.005931 1.358109  63 1 0 -5.486086 4.133094 -0.468594  64 1 0 5.457492 2.173664 1.039629  65 1 0 -3.742453 4.315208 -2.291246  66 1 0 5.485260 -4.130746 -0.485273  67 1 0 5.454537 -2.183816 1.030921  68 1 0 2.018726 -2.472544 -2.467663  69 1 0 3.746894 4.311166 -2.291001  70 1 0 5.490456 4.126860 -0.468516 | 1 29 0 -1.388410 0.003480 -1.088128  2 29 0 1.388072 0.001928 -1.088253  3 8 0 -0.000913 -1.272142 -1.647557  4 8 0 0.000528 1.280209 -1.641359  5 7 0 -2.804547 -1.392234 -0.885257  6 7 0 -1.174023 -0.001165 1.172417  7 7 0 -2.802982 1.399860 -0.879673  8 7 0 2.802628 -1.395412 -0.885669  9 7 0 2.804254 1.396688 -0.879745  10 7 0 1.173873 -0.002586 1.172314  11 6 0 -4.705345 2.257246 0.257128  12 6 0 -2.406562 -0.004840 3.237170  13 6 0 -2.338434 -0.001824 1.815943  14 6 0 3.762042 -3.464024 -1.615538  15 6 0 -1.243914 -0.007238 3.970750  16 6 0 0.000015 -0.006595 3.299734  17 6 0 -3.726429 1.266874 0.092474  18 6 0 2.406587 -0.008040 3.236959  19 6 0 1.244001 -0.008971 3.970640  20 6 0 -0.000045 -0.003395 1.872558  21 6 0 -4.878741 -0.000593 1.933859  22 6 0 2.813547 2.458476 -1.705970  23 6 0 3.665772 -0.003786 0.984966  24 6 0 -2.813937 -2.450684 -1.715758  25 6 0 -2.811146 2.461543 -1.706043  26 6 0 2.338339 -0.004742 1.815738  27 6 0 -4.726278 -3.355162 -0.608279  28 6 0 -4.707512 -2.252247 0.248588  29 6 0 -3.766364 -3.459853 -1.614774  30 6 0 -3.727718 -1.262154 0.087565  31 6 0 4.878652 -0.006505 1.933436  32 6 0 3.727628 1.262540 0.092314  33 6 0 3.726037 -1.266488 0.087079  34 6 0 -4.722877 3.363500 -0.595457  35 6 0 4.707666 2.251791 0.257033  36 6 0 -3.665938 0.000593 0.985286  37 6 0 -3.762624 3.471224 -1.601314  38 6 0 4.722177 -3.360530 -0.609131  39 6 0 4.704737 -2.257700 0.247872  40 6 0 2.810743 -2.453768 -1.716303  41 6 0 3.766166 3.467072 -1.601176  42 6 0 4.726375 3.358139 -0.595407  43 1 0 -0.001293 -2.056715 -1.074682  44 1 0 0.000962 2.061942 -1.064608  45 1 0 -5.456107 2.179202 1.039084  46 1 0 -3.361356 -0.005334 3.754111  47 1 0 3.742381 -4.305651 -2.308533  48 1 0 -1.270090 -0.009619 5.062157  49 1 0 3.361425 -0.009866 3.753815  50 1 0 1.270270 -0.011521 5.062044  51 1 0 -4.875577 0.884697 2.581453  52 1 0 -4.876426 -0.888271 2.578156  53 1 0 -5.813593 0.000902 1.358603  54 1 0 2.021330 2.482648 -2.456562  55 1 0 -2.021967 -2.471702 -2.466708  56 1 0 -2.018951 2.484723 -2.456690  57 1 0 -5.491400 -4.123870 -0.484570  58 1 0 -5.458033 -2.176543 1.031012  59 1 0 -3.747725 -4.301587 -2.307666  60 1 0 4.875404 -0.894324 2.577539  61 1 0 4.876523 0.878644 2.581221  62 1 0 5.813458 -0.005927 1.358103  63 1 0 -5.487297 4.132448 -0.468930  64 1 0 5.458394 2.172791 1.038924  65 1 0 -3.743030 4.315659 -2.290884  66 1 0 5.486451 -4.130108 -0.485587  67 1 0 5.455429 -2.182945 1.030226  68 1 0 2.018666 -2.473801 -2.467167  69 1 0 3.747471 4.311623 -2.290629  70 1 0 5.491664 4.126217 -0.468841 |

| Doublet 1^3+^ | Quartet 1^3+^ |
| --- | --- |
| 1 29 0 -1.450318 0.003877 -1.057308  2 29 0 1.380564 0.001029 -0.998698  3 8 0 -0.122381 -1.233690 -1.463626  4 8 0 -0.120063 1.241125 -1.456586  5 7 0 -2.794110 -1.324656 -0.924792  6 7 0 -1.168034 -0.002352 1.213518  7 7 0 -2.791676 1.334208 -0.915843  8 7 0 2.772505 -1.387020 -0.879795  9 7 0 2.774226 1.386941 -0.876179  10 7 0 1.174976 -0.002553 1.205806  11 6 0 -4.602076 2.281501 0.263480  12 6 0 -2.399776 -0.006472 3.280998  13 6 0 -2.326643 -0.003500 1.864252  14 6 0 3.709095 -3.456647 -1.625033  15 6 0 -1.232454 -0.007618 4.010874  16 6 0 0.009983 -0.006014 3.336602  17 6 0 -3.672483 1.248023 0.102521  18 6 0 2.419039 -0.005150 3.260656  19 6 0 1.259059 -0.006547 4.000125  20 6 0 0.006715 -0.003641 1.910585  21 6 0 -4.873600 -0.003125 1.924543  22 6 0 2.768858 2.440806 -1.713469  23 6 0 3.658118 -0.002958 0.990411  24 6 0 -2.798240 -2.331714 -1.820308  25 6 0 -2.793888 2.347319 -1.804555  26 6 0 2.344502 -0.003403 1.840923  27 6 0 -4.623285 -3.342492 -0.653370  28 6 0 -4.603828 -2.278237 0.250385  29 6 0 -3.718394 -3.367743 -1.714245  30 6 0 -3.673782 -1.244323 0.095024  31 6 0 4.884276 -0.004881 1.920709  32 6 0 3.704088 1.261488 0.093187  33 6 0 3.702228 -1.265371 0.090223  34 6 0 -4.619787 3.351595 -0.633393  35 6 0 4.676512 2.258418 0.243626  36 6 0 -3.636150 -0.000921 1.011305  37 6 0 -3.713368 3.383334 -1.692769  38 6 0 4.674542 -3.364080 -0.622778  39 6 0 4.672763 -2.264388 0.238905  40 6 0 2.765666 -2.439033 -1.719393  41 6 0 3.714242 3.456431 -1.617366  42 6 0 4.679983 3.359906 -0.615762  43 1 0 -0.171756 -2.008250 -0.871963  44 1 0 -0.167132 2.012097 -0.860063  45 1 0 -5.315546 2.253747 1.082991  46 1 0 -3.355317 -0.007701 3.797246  47 1 0 3.679938 -4.294291 -2.322159  48 1 0 -1.255948 -0.009672 5.102099  49 1 0 3.376878 -0.005519 3.772217  50 1 0 1.291499 -0.008063 5.091082  51 1 0 -4.879781 0.880098 2.574576  52 1 0 -4.880075 -0.889811 2.569836  53 1 0 -5.794580 -0.001394 1.327329  54 1 0 1.981693 2.454629 -2.469603  55 1 0 -2.047214 -2.291936 -2.609785  56 1 0 -2.041993 2.312101 -2.593436  57 1 0 -5.354024 -4.143059 -0.527371  58 1 0 -5.316255 -2.255414 1.070964  59 1 0 -3.710924 -4.169818 -2.452239  60 1 0 4.887984 -0.891998 2.565658  61 1 0 4.889274 0.880874 2.567528  62 1 0 5.811150 -0.004946 1.332998  63 1 0 -5.350294 4.151699 -0.503220  64 1 0 5.433263 2.188437 1.020537  65 1 0 -3.704349 4.190293 -2.425399  66 1 0 5.433224 -4.140089 -0.506829  67 1 0 5.429311 -2.197465 1.016294  68 1 0 1.978903 -2.449679 -2.476006  69 1 0 3.686360 4.295620 -2.312685  70 1 0 5.440184 4.134236 -0.498561 | 1 29 0 -1.405652 0.003444 -0.933930  2 29 0 1.405328 0.001837 -0.934051  3 8 0 -0.000892 -1.245253 -1.424952  4 8 0 0.000529 1.252629 -1.419549  5 7 0 -2.827293 -1.382637 -0.855622  6 7 0 -1.192597 -0.001260 1.163270  7 7 0 -2.825628 1.390768 -0.849255  8 7 0 2.825297 -1.385974 -0.855778  9 7 0 2.826994 1.387423 -0.849608  10 7 0 1.192442 -0.002631 1.163165  11 6 0 -4.761924 2.235210 0.231940  12 6 0 -2.409323 -0.004500 3.227488  13 6 0 -2.362089 -0.001806 1.810947  14 6 0 3.803154 -3.411275 -1.656187  15 6 0 -1.237430 -0.006712 3.942189  16 6 0 0.000012 -0.006209 3.262105  17 6 0 -3.765194 1.260880 0.105828  18 6 0 2.409345 -0.007537 3.227275  19 6 0 1.237513 -0.008361 3.942080  20 6 0 -0.000049 -0.003270 1.832009  21 6 0 -4.900328 -0.001076 1.965392  22 6 0 2.833966 2.422590 -1.708550  23 6 0 3.698894 -0.003887 1.003939  24 6 0 -2.834163 -2.414239 -1.718855  25 6 0 -2.831422 2.425989 -1.708141  26 6 0 2.361989 -0.004599 1.810739  27 6 0 -4.781060 -3.312098 -0.661903  28 6 0 -4.763129 -2.230866 0.223447  29 6 0 -3.807542 -3.406827 -1.655904  30 6 0 -3.766183 -1.256345 0.100623  31 6 0 4.900234 -0.006787 1.964970  32 6 0 3.766386 1.256543 0.105516  33 6 0 3.764505 -1.260683 0.100281  34 6 0 -4.778861 3.320066 -0.648995  35 6 0 4.764144 2.229814 0.231672  36 6 0 -3.699064 0.000389 1.004262  37 6 0 -3.804504 3.418644 -1.641800  38 6 0 4.777007 -3.317546 -0.662420  39 6 0 4.760433 -2.236271 0.222904  40 6 0 2.830850 -2.417622 -1.718964  41 6 0 3.808124 3.414188 -1.642188  42 6 0 4.782292 3.314624 -0.649296  43 1 0 -0.001298 -2.096203 -0.951637  44 1 0 0.000989 2.101721 -0.942906  45 1 0 -5.525853 2.159683 1.001402  46 1 0 -3.359696 -0.004841 3.751364  47 1 0 3.785812 -4.235319 -2.369699  48 1 0 -1.248354 -0.008815 5.033344  49 1 0 3.359763 -0.009126 3.751066  50 1 0 1.248530 -0.010633 5.033233  51 1 0 -4.894132 0.886530 2.609696  52 1 0 -4.894458 -0.890893 2.606626  53 1 0 -5.837238 0.000107 1.394415  54 1 0 2.035693 2.442448 -2.452288  55 1 0 -2.036347 -2.430469 -2.463166  56 1 0 -2.033087 2.444985 -2.451838  57 1 0 -5.558804 -4.072213 -0.570211  58 1 0 -5.526449 -2.158267 0.993803  59 1 0 -3.791262 -4.230862 -2.369451  60 1 0 4.893462 -0.896733 2.606022  61 1 0 4.895037 0.880689 2.609454  62 1 0 5.837098 -0.006495 1.393918  63 1 0 -5.556475 4.080009 -0.554818  64 1 0 5.527933 2.153489 1.001193  65 1 0 -3.787405 4.245646 -2.351886  66 1 0 5.553967 -4.078481 -0.570878  67 1 0 5.524009 -2.164481 0.993082  68 1 0 2.032860 -2.432980 -2.463111  69 1 0 3.791974 4.241178 -2.352310  70 1 0 5.560694 4.073755 -0.555089 |

| Doublet 1-H^2+^ | Quartet 1-H^2+^ |
| --- | --- |
| 1 29 0 -1.321723 0.080597 -1.057565  2 29 0 1.398009 0.133269 -1.120404  3 8 0 0.114823 -1.034298 -1.586354  4 8 0 0.103153 1.444500 -1.528009  5 7 0 -2.663530 -1.386097 -0.860353  6 7 0 -1.171420 0.095869 1.188824  7 7 0 -2.793396 1.420628 -0.872272  8 7 0 2.658273 -1.304745 -0.930798  9 7 0 2.826061 1.432572 -0.872376  10 7 0 1.167134 0.098382 1.191876  11 6 0 -4.778408 2.184129 0.190659  12 6 0 -2.413871 0.067836 3.243430  13 6 0 -2.338413 0.059835 1.821986  14 6 0 3.413175 -3.443822 -1.665105  15 6 0 -1.253685 0.091117 3.982839  16 6 0 -0.004524 0.096073 3.318781  17 6 0 -3.745872 1.244017 0.064354  18 6 0 2.404983 0.055317 3.251935  19 6 0 1.241745 0.083949 3.987361  20 6 0 -0.003301 0.103036 1.892072  21 6 0 -4.875034 -0.064304 1.894325  22 6 0 2.866700 2.496864 -1.697252  23 6 0 3.633988 -0.012158 0.973447  24 6 0 -2.571092 -2.470678 -1.648973  25 6 0 -2.817906 2.486280 -1.693527  26 6 0 2.328040 0.055255 1.832075  27 6 0 -4.450981 -3.466253 -0.563796  28 6 0 -4.532042 -2.337642 0.255648  29 6 0 -3.453578 -3.540822 -1.536140  30 6 0 -3.615236 -1.289883 0.088592  31 6 0 4.866078 -0.106794 1.889631  32 6 0 3.744787 1.254683 0.097214  33 6 0 3.580542 -1.257072 0.054049  34 6 0 -4.813839 3.291958 -0.659403  35 6 0 4.758569 2.207041 0.263305  36 6 0 -3.647349 -0.010173 0.968052  37 6 0 -3.817575 3.451378 -1.622746  38 6 0 4.375321 -3.433465 -0.655920  39 6 0 4.456588 -2.339531 0.208224  40 6 0 2.555687 -2.353917 -1.765347  41 6 0 3.856217 3.466886 -1.585456  42 6 0 4.813738 3.317047 -0.582299  43 1 0 0.151443 2.154866 -0.862188  44 1 0 -5.555811 2.066071 0.941097  45 1 0 -3.371633 0.052257 3.755504  46 1 0 3.316196 -4.276228 -2.362356  47 1 0 -1.286318 0.095033 5.074068  48 1 0 3.361842 0.032185 3.765554  49 1 0 1.270623 0.085988 5.078715  50 1 0 -4.928976 0.828673 2.529252  51 1 0 -4.835901 -0.941978 2.551337  52 1 0 -5.799043 -0.120023 1.304154  53 1 0 2.074748 2.553121 -2.445471  54 1 0 -1.756275 -2.460823 -2.375371  55 1 0 -2.000471 2.551410 -2.414325  56 1 0 -5.166593 -4.280586 -0.435234  57 1 0 -5.308154 -2.288858 1.015134  58 1 0 -3.354668 -4.404687 -2.194185  59 1 0 4.813509 -0.996170 2.529423  60 1 0 4.934217 0.770421 2.544371  61 1 0 5.786152 -0.163142 1.293377  62 1 0 -5.618967 4.022897 -0.563537  63 1 0 5.506983 2.092187 1.042940  64 1 0 -3.808470 4.300971 -2.306173  65 1 0 5.063987 -4.271315 -0.532997  66 1 0 5.202625 -2.339128 0.998468  67 1 0 1.748702 -2.292576 -2.496557  68 1 0 3.866486 4.314382 -2.271032  69 1 0 5.606258 4.056431 -0.454021 | 1 29 0 -1.356786 0.083965 -1.035306  2 29 0 1.356568 0.082384 -1.035426  3 8 0 -0.000814 -1.084074 -1.672345  4 8 0 0.000658 1.447417 -1.487961  5 7 0 -2.726318 -1.372521 -0.883862  6 7 0 -1.177068 0.037607 1.160643  7 7 0 -2.833060 1.433296 -0.852084  8 7 0 2.724432 -1.375691 -0.884140  9 7 0 2.834408 1.429992 -0.852286  10 7 0 1.177003 0.036188 1.160546  11 6 0 -4.816608 2.179097 0.224193  12 6 0 -2.407651 0.079692 3.223466  13 6 0 -2.343821 0.035977 1.803989  14 6 0 3.572067 -3.489247 -1.615762  15 6 0 -1.243355 0.114353 3.953474  16 6 0 0.000093 0.098573 3.281726  17 6 0 -3.779778 1.245944 0.086617  18 6 0 2.407808 0.076563 3.223270  19 6 0 1.243614 0.112659 3.953374  20 6 0 0.000011 0.060721 1.855634  21 6 0 -4.880451 -0.077356 1.922611  22 6 0 2.866595 2.498520 -1.668582  23 6 0 3.668967 -0.023377 0.974846  24 6 0 -2.667594 -2.435129 -1.705258  25 6 0 -2.864121 2.501823 -1.668427  26 6 0 2.343807 0.033095 1.803795  27 6 0 -4.560547 -3.417875 -0.629261  28 6 0 -4.606716 -2.312335 0.223472  29 6 0 -3.576485 -3.485110 -1.615348  30 6 0 -3.668867 -1.281234 0.074065  31 6 0 4.880323 -0.083078 1.922164  32 6 0 3.780954 1.241562 0.086367  33 6 0 3.667186 -1.285516 0.073691  34 6 0 -4.861781 3.289894 -0.621854  35 6 0 4.818793 2.173594 0.223960  36 6 0 -3.669122 -0.019056 0.975166  37 6 0 -3.870985 3.459016 -1.589209  38 6 0 4.556306 -3.423177 -0.629773  39 6 0 4.603849 -2.317711 0.222982  40 6 0 2.664387 -2.438209 -1.705554  41 6 0 3.874494 3.454620 -1.589354  42 6 0 4.865132 3.284387 -0.622032  43 1 0 0.001083 2.159538 -0.825085  44 1 0 -5.589135 2.053473 0.978425  45 1 0 -3.361745 0.085898 3.741542  46 1 0 3.499958 -4.334146 -2.301288  47 1 0 -1.267336 0.147836 5.044267  48 1 0 3.361946 0.081488 3.741276  49 1 0 1.267728 0.145986 5.044168  50 1 0 -4.925783 0.812197 2.562591  51 1 0 -4.832127 -0.960314 2.571971  52 1 0 -5.812768 -0.127759 1.345759  53 1 0 2.048213 2.572609 -2.386974  54 1 0 -1.854921 -2.426271 -2.432989  55 1 0 -2.045682 2.574993 -2.386849  56 1 0 -5.292811 -4.219512 -0.515932  57 1 0 -5.372299 -2.267913 0.993776  58 1 0 -3.505434 -4.330108 -2.300863  59 1 0 4.831092 -0.966027 2.571461  60 1 0 4.926688 0.806377 2.562209  61 1 0 5.812529 -0.134454 1.345214  62 1 0 -5.670189 4.016236 -0.519150  63 1 0 5.591211 2.047102 0.978157  64 1 0 -3.870363 4.311777 -2.268720  65 1 0 5.287646 -4.225671 -0.516535  66 1 0 5.369554 -2.274212 0.993219  67 1 0 1.851647 -2.428389 -2.433197  68 1 0 3.874772 4.307409 -2.268827  69 1 0 5.674322 4.009856 -0.519321 |

| Doublet TS-1^3+^ |
| --- |
| 1 29 0 -1.484885 -0.072072 -1.041541  2 29 0 1.370270 -0.142512 -1.020397  3 8 0 -0.088264 -1.376794 -1.380058  4 8 0 -0.041039 1.099437 -1.893731  5 7 0 -2.898429 -1.437890 -0.740084  6 7 0 -1.234163 0.140504 1.168674  7 7 0 -2.891394 1.327296 -1.014802  8 7 0 2.709689 -1.595169 -0.740317  9 7 0 2.852993 1.179778 -0.959270  10 7 0 1.119894 0.061887 1.179875  11 6 0 -4.744155 2.327110 0.089732  12 6 0 -2.466649 0.263997 3.232880  13 6 0 -2.399309 0.183272 1.816012  14 6 0 3.562954 -3.757470 -1.298339  15 6 0 -1.304837 0.281847 3.965447  16 6 0 -0.063520 0.209417 3.295826  17 6 0 -3.786640 1.308072 -0.005273  18 6 0 2.338109 0.093889 3.255360  19 6 0 1.173458 0.191540 3.977007  20 6 0 -0.059189 0.140847 1.870144  21 6 0 -4.939428 0.228390 1.948093  22 6 0 2.958645 2.161007 -1.875220  23 6 0 3.613988 -0.106927 1.030927  24 6 0 -2.912296 -2.567838 -1.469865  25 6 0 -2.935332 2.288855 -1.955366  26 6 0 2.279766 0.024423 1.837307  27 6 0 -4.834786 -3.344812 -0.285224  28 6 0 -4.806490 -2.167936 0.467088  29 6 0 -3.874481 -3.553307 -1.275071  30 6 0 -3.816580 -1.207538 0.218077  31 6 0 4.814873 -0.097617 1.994894  32 6 0 3.751247 1.076741 0.043313  33 6 0 3.624441 -1.439070 0.235498  34 6 0 -4.782485 3.339848 -0.870338  35 6 0 4.782567 2.019686 0.150490  36 6 0 -3.731777 0.131207 0.997713  37 6 0 -3.869634 3.317696 -1.923886  38 6 0 4.515312 -3.627194 -0.287610  39 6 0 4.547055 -2.464015 0.485559  40 6 0 2.666708 -2.710942 -1.490148  41 6 0 3.965367 3.118500 -1.829516  42 6 0 4.888769 3.047628 -0.787752  43 1 0 -0.111370 -2.107024 -0.738879  44 1 0 -0.014197 2.033676 -1.610817  45 1 0 -5.465703 2.340152 0.902095  46 1 0 -3.420953 0.308464 3.747951  47 1 0 3.507803 -4.646741 -1.926710  48 1 0 -1.329213 0.342155 5.054980  49 1 0 3.287635 0.069341 3.780432  50 1 0 1.191804 0.246592 5.066943  51 1 0 -4.916296 1.161722 2.523329  52 1 0 -4.950723 -0.605697 2.659950  53 1 0 -5.875731 0.201104 1.376058  54 1 0 2.213012 2.171685 -2.668918  55 1 0 -2.122802 -2.666321 -2.216761  56 1 0 -2.200767 2.219084 -2.757131  57 1 0 -5.607929 -4.091017 -0.093485  58 1 0 -5.556738 -2.013311 1.237832  59 1 0 -3.863047 -4.456039 -1.886272  60 1 0 4.762781 -0.937818 2.697795  61 1 0 4.846605 0.829819 2.579231  62 1 0 5.754230 -0.181628 1.433556  63 1 0 -5.529724 4.131717 -0.794122  64 1 0 5.508611 1.961829 0.956716  65 1 0 -3.871881 4.075304 -2.707714  66 1 0 5.236369 -4.423840 -0.095767  67 1 0 5.290838 -2.370251 1.272287  68 1 0 1.884826 -2.748132 -2.250572  69 1 0 4.014192 3.894128 -2.593918  70 1 0 5.693837 3.779565 -0.702576  71 6 0 -1.526265 -0.573118 -5.178224  72 6 0 -2.770417 -0.800351 -5.745095  73 6 0 -3.523521 0.279696 -6.247885  74 6 0 -3.019763 1.594149 -6.181700  75 6 0 -1.777245 1.831956 -5.614842  76 6 0 -0.989581 0.750797 -5.110123  77 1 0 -0.935410 -1.405193 -4.789681  78 1 0 -3.168644 -1.814855 -5.805421  79 1 0 -4.504428 0.096748 -6.691639  80 1 0 -3.608765 2.422555 -6.579456  81 1 0 -1.378173 2.847445 -5.563988  82 6 0 0.297088 0.993419 -4.512365  83 1 0 0.073316 1.086988 -3.231430  84 1 0 0.990993 0.142885 -4.483502  85 1 0 0.765795 1.958179 -4.741908 |

| Doublet TS-1-H^2+^ | Quartet TS-1-H^2+^ |
| --- | --- |
| 1 29 0 -1.660927 0.055647 -1.037529  2 29 0 1.037755 -0.135879 -1.086194  3 8 0 -0.440423 -1.433185 -1.345056  4 8 0 -0.307211 0.984098 -1.961578  5 7 0 -3.227235 -1.105056 -0.654611  6 7 0 -1.340079 0.159181 1.258963  7 7 0 -2.780089 1.661334 -0.807516  8 7 0 2.438506 -1.576880 -0.935419  9 7 0 2.468811 1.264383 -0.870862  10 7 0 0.981847 -0.083736 1.169782  11 6 0 -4.408788 2.885296 0.414088  12 6 0 -2.505707 0.148340 3.361694  13 6 0 -2.474037 0.257164 1.942966  14 6 0 3.303878 -3.652532 -1.756967  15 6 0 -1.333238 -0.061377 4.049988  16 6 0 -0.115608 -0.149297 3.336168  17 6 0 -3.654255 1.719115 0.219922  18 6 0 2.276919 -0.378415 3.171622  19 6 0 1.143573 -0.340699 3.950584  20 6 0 -0.159738 -0.029408 1.915142  21 6 0 -4.962355 0.733404 2.132799  22 6 0 2.406283 2.399718 -1.589533  23 6 0 3.449761 -0.224524 0.872808  24 6 0 -3.410960 -2.193836 -1.423299  25 6 0 -2.631375 2.697584 -1.652761  26 6 0 2.163032 -0.232203 1.760948  27 6 0 -5.451278 -2.693679 -0.286129  28 6 0 -5.244971 -1.564571 0.510011  29 6 0 -4.519504 -3.020915 -1.271506  30 6 0 -4.107754 -0.770567 0.306272  31 6 0 4.703198 -0.262042 1.764964  32 6 0 3.461350 1.071615 0.023546  33 6 0 3.446906 -1.465184 -0.052140  34 6 0 -4.263882 3.966119 -0.457608  35 6 0 4.453318 2.049902 0.182841  36 6 0 -3.801644 0.489678 1.151681  37 6 0 -3.362718 3.872780 -1.516799  38 6 0 4.363710 -3.554950 -0.854797  39 6 0 4.435408 -2.457969 0.007071  40 6 0 2.354383 -2.635273 -1.761550  41 6 0 3.360069 3.406634 -1.478229  42 6 0 4.407048 3.220333 -0.577244  43 1 0 -0.486156 -2.064270 -0.604785  44 1 0 -5.112073 2.961979 1.238871  45 1 0 -3.439141 0.225053 3.911578  46 1 0 3.208215 -4.494451 -2.443331  47 1 0 -1.332024 -0.152593 5.137935  48 1 0 3.244626 -0.515097 3.645231  49 1 0 1.207260 -0.445905 5.035323  50 1 0 -4.757561 1.595412 2.779960  51 1 0 -5.122933 -0.138663 2.777756  52 1 0 -5.894536 0.924237 1.585517  53 1 0 1.546689 2.489009 -2.252480  54 1 0 -2.633565 -2.389981 -2.164093  55 1 0 -1.886596 2.558500 -2.434635  56 1 0 -6.337795 -3.311302 -0.130164  57 1 0 -5.971385 -1.316912 1.279767  58 1 0 -4.641624 -3.894444 -1.912551  59 1 0 4.733527 -1.180515 2.363737  60 1 0 4.723104 0.591303 2.454394  61 1 0 5.612652 -0.230102 1.151234  62 1 0 -4.855624 4.870316 -0.303109  63 1 0 5.261762 1.914196 0.896265  64 1 0 -3.217276 4.688859 -2.225137  65 1 0 5.133830 -4.327622 -0.813437  66 1 0 5.258822 -2.390507 0.713545  67 1 0 1.488221 -2.650523 -2.425783  68 1 0 3.273406 4.309363 -2.083656  69 1 0 5.182055 3.979104 -0.453559  70 6 0 -0.144655 -0.946729 -5.399197  71 6 0 -1.013262 -1.793611 -6.085495  72 6 0 -2.251159 -1.316188 -6.542398  73 6 0 -2.611057 0.019965 -6.309305  74 6 0 -1.745396 0.870593 -5.624062  75 6 0 -0.487477 0.408924 -5.156568  76 1 0 0.820475 -1.321950 -5.048747  77 1 0 -0.727067 -2.831768 -6.270387  78 1 0 -2.930646 -1.981171 -7.080471  79 1 0 -3.571723 0.396724 -6.668536  80 1 0 -2.028977 1.911680 -5.448368  81 6 0 0.395387 1.278279 -4.395331  82 1 0 -0.019816 1.085870 -3.126791  83 1 0 1.448900 0.977657 -4.352158  84 1 0 0.247173 2.357091 -4.526233 | 1 29 0 -1.999870 -0.508038 -0.737080  2 29 0 0.659127 -0.193947 -1.231479  3 8 0 -0.554530 -1.747851 -1.250987  4 8 0 -0.952771 0.722333 -1.818513  5 7 0 -3.218497 -1.978543 -0.098617  6 7 0 -1.457052 -0.145347 1.417250  7 7 0 -3.490770 0.804666 -0.454769  8 7 0 2.321414 -1.323997 -1.120045  9 7 0 1.818410 1.432493 -1.427481  10 7 0 0.836062 0.123497 0.996065  11 6 0 -5.245144 1.680207 0.890372  12 6 0 -2.308848 -0.128510 3.665256  13 6 0 -2.487420 -0.216569 2.256767  14 6 0 3.501198 -3.288389 -1.813993  15 6 0 -1.045189 0.042646 4.178696  16 6 0 0.055621 0.135466 3.296998  17 6 0 -4.233960 0.734404 0.667299  18 6 0 2.393985 0.422484 2.802610  19 6 0 1.386286 0.328090 3.732847  20 6 0 -0.189425 0.033981 1.894778  21 6 0 -4.964097 -0.356348 2.814461  22 6 0 1.477789 2.385472 -2.313122  23 6 0 3.232323 0.439957 0.363288  24 6 0 -3.238288 -3.145108 -0.767923  25 6 0 -3.703329 1.770399 -1.366152  26 6 0 2.083092 0.319017 1.418206  27 6 0 -4.930953 -3.998197 0.685386  28 6 0 -4.897428 -2.785768 1.378427  29 6 0 -4.087296 -4.187715 -0.409354  30 6 0 -4.021964 -1.772886 0.961568  31 6 0 4.561941 0.761974 1.068787  32 6 0 2.901694 1.575524 -0.638296  33 6 0 3.376549 -0.900759 -0.399549  34 6 0 -5.480046 2.680922 -0.055209  35 6 0 3.686919 2.732961 -0.739673  36 6 0 -3.928608 -0.401659 1.676461  37 6 0 -4.697832 2.730715 -1.208531  38 6 0 4.609187 -2.869808 -1.076870  39 6 0 4.547709 -1.671057 -0.362250  40 6 0 2.368273 -2.480451 -1.805404  41 6 0 2.221550 3.551205 -2.468910  42 6 0 3.346039 3.724125 -1.662831  43 1 0 -0.347438 -2.356913 -0.521709  44 1 0 -5.852657 1.649481 1.790942  45 1 0 -3.154151 -0.194528 4.343322  46 1 0 3.506724 -4.218003 -2.383890  47 1 0 -0.883032 0.112017 5.256037  48 1 0 3.415026 0.574855 3.138829  49 1 0 1.600946 0.405526 4.800472  50 1 0 -4.911829 0.594412 3.359487  51 1 0 -4.797705 -1.167543 3.533555  52 1 0 -5.979175 -0.465300 2.411206  53 1 0 0.573401 2.192102 -2.890304  54 1 0 -2.538918 -3.224553 -1.602279  55 1 0 -3.032560 1.762853 -2.225697  56 1 0 -5.614086 -4.786945 1.006244  57 1 0 -5.553265 -2.645417 2.233805  58 1 0 -4.080578 -5.119097 -0.976363  59 1 0 4.844998 -0.037819 1.763963  60 1 0 4.491067 1.696342 1.639241  61 1 0 5.368163 0.870744 0.332065  62 1 0 -6.269442 3.415084 0.116794  63 1 0 4.560878 2.874066 -0.109204  64 1 0 -4.844766 3.495604 -1.971649  65 1 0 5.521076 -3.469416 -1.051802  66 1 0 5.412381 -1.352286 0.214136  67 1 0 1.458120 -2.748362 -2.345406  68 1 0 1.917685 4.298399 -3.202654  69 1 0 3.959624 4.623405 -1.743886  70 6 0 -0.670411 1.671961 -5.769002  71 6 0 0.254543 2.291279 -6.610865  72 6 0 1.501950 1.695470 -6.846288  73 6 0 1.815585 0.472979 -6.235124  74 6 0 0.891637 -0.147222 -5.392812  75 6 0 -0.372346 0.437539 -5.146507  76 1 0 -1.642557 2.139357 -5.590542  77 1 0 0.003721 3.241091 -7.089196  78 1 0 2.225592 2.180432 -7.505640  79 1 0 2.784179 0.002378 -6.420038  80 1 0 1.139552 -1.101973 -4.921567  81 6 0 -1.332915 -0.197533 -4.227632  82 1 0 -1.129122 0.249863 -3.094640  83 1 0 -2.382505 0.074465 -4.402440  84 1 0 -1.199687 -1.278354 -4.09652 |
